# Effectiveness of Blended Learning in Nursing Education

**DOI:** 10.3390/ijerph17051589

**Published:** 2020-03-01

**Authors:** María Consuelo Sáiz-Manzanares, María-Camino Escolar-Llamazares, Álvar Arnaiz González

**Affiliations:** 1 Departamento de Ciencias de la Salud, Facultad de Ciencias de la Salud, Universidad de Burgos, C/ Comendadores s/n, 09001 Burgos, Spain; cescolar@ubu.es; 2 Departamento de Ingeniería Informática, Escuela Politécnica Superior, Universidad de Burgos, Avda. Cantabria s/n, 09006 Burgos, Spain; alvarag@ubu.es

**Keywords:** learning management system, higher education, nursing, data mining

## Abstract

Currently, teaching in higher education is being heavily developed by learning management systems that record the learning behaviour of both students and teachers. The use of learning management systems that include project-based learning and hypermedia resources increases safer learning, and it is proven to be effective in degrees such as nursing. In this study, we worked with 120 students in the third year of nursing degree. Two types of blended learning were applied (more interaction in learning management systems with hypermedia resources vs. none). Supervised learning techniques were applied: linear regression and k-means clustering. The results indicated that the type of blended learning in use predicted 40.4% of student learning outcomes. It also predicted 71.9% of the effective learning behaviors of students in learning management systems. It therefore appears that blended learning applied in Learning Management System (LMS) with hypermedia resources favors greater achievement of effective learning. Likewise, with this type of Blended Learning (BL) a larger number of students were found to belong to the intermediate cluster, suggesting that this environment strengthens better results in a larger number of students. BL with hypermedia resources and project-based learning increase students´ learning outcomes and interaction in learning management systems. Future research will be aimed at verifying these results in other nursing degree courses.

## 1. Introduction

In approximately the last decade there has been a marked interest in investigating ways of teaching other than traditional face-to-face. The incorporation of technological resources such as virtual platforms and hypermedia resources, combined with other innovative, methodological techniques such as project-based or problem-based learning, have revolutionized the teaching–learning process. The aim is to teach in the most efficient way possible and to make the most of resources while ensuring sustainability. These technological and methodological resources have been applied to different disciplines, especially in the field of health sciences (medicine, pharmacy, psychology, veterinary, etc.). However, in recent years these resources have been incorporated into nursing studies. Next, an approach will be made for the most relevant concepts of teaching in a virtual platform, which has been called blended teaching, as well as the implementation of methodological resources for project-based learning. Likewise, special importance will be given to its application for the formation of future nursing programs by analyzing the pros and cons of this form of teaching and learning in the society of the 21st century. For this reason, the most relevant concepts of these new forms of teaching and their specific application to the nursing degree will be dealt with below. The final objective of this work is to study the effectiveness of different blended learning environments in the teaching of future nurses.

Twenty-first century society requires students and graduates to develop a series of skills related to two important leitmotifs: collaborative work and operation of information and communications technology (ICT). It is increasingly necessary to possess effective and rapid problem-solving skills and to develop digital competences [1]. The use of learning management systems (LMS) is, therefore, a reference in instructional practice, especially in higher education, as is the implementation of collaborative work in these methodological settings for the resolution of tasks and problems. A good example might be the use of project-based learning (PBL) methodology [2]. Recent investigations have confirmed that if such a methodology is accompanied by the use of hypermedia resources (e.g., flipped learning experiences, quizzes, use of wikis, on-line glossaries, etc.), then acquisition of deep learning is strengthened in students [3]. Deep learning is a concept developed in the framework of the taxonomy of Bloom [4]. It corresponds to the highest level of learning competences (comprehending, analyzing, summarizing, and evaluating their own learning). One of the currents of thought in LMS learning environments suggests that learning in these environments implies deeper learning from the point of view of cognitive and metacognitive complexity, as these facilitate self-regulated learning (SRL) and meaningful learning [5].

Likewise, LMS permit a more precise analysis of interactions which are logged in records (or logs). The logs represent units of information and registration that stores precise data on the frequency of user interactions and their duration [6]. LMS also facilitates the inclusion of hypermedia resources [7]. The use of these resources is especially relevant in health science degrees (nursing, medicine, pharmacy, etc.) since it implements practical assumptions in the work, which has been proven to reduce errors in the workplace [8].

There are various stages in this instruction process that will facilitate or inhibit the efficiency and depth of the learning process. One of them is the design of learning tasks in LMS [9,10]. Another essential element is that the teacher plans for process-oriented feedback [11].

### 1.1. Teaching through Learning Management Systems

The teacher has to reflect, among other points, on the following points: (1) the aims of the subject module, (2) to whom it is addressed, (3) what previous knowledge is required for a successful approach to the subject matter, (4) the type of learning tasks that facilitate content acquisition, (5) the metacognitive skills of the students prior to the instruction, (6) the cognitive and the metacognitive skills in each task needed for its effective solution, and (7) when and where the teaching–learning process will be developed. Likewise, the teacher has to plan follow-up with both the individual student and the group behavior on the platform. As has been argued, solving problems in a collaborative way is one of the most demanding skills in 21st century society. These types of competences are key references in educational and technological areas and for entry into employment. Collaborative work facilitates the construction of deep and effective learning in the students [10]. A scheme for the preparation of pedagogic design in the LMS may be seen in Table 1.

Nevertheless, computational techniques are required to conduct a conclusive analysis of student behavior in LMS. As previously mentioned, at present a broad percentage of learning is done in virtual environments, in what is called blended learning teaching. A lot of data can be recorded by LMS and accessed through logs. However, educational data mining (EDM) [12,13] is needed to study them precisely. Machine learning techniques can be applied to EDM. Subsequently, possible applications of those techniques will be presented in the analysis of learning data in LMS environments.

### 1.2. Application of Artificial Intelligence Techniques to Analyze the Teaching and Learning Process

Development of the internet and information and communications technology (ICT) has expanded learner access to information, and they have changed the way that information is taught and the way it is learned [14]. A learning management system (LMS) is an interactive learning environment that facilitates both teaching and learning. In addition, these software environments record all the actions performed by the teacher and by the students, under individual and group headings. However, those logs store a lot of data and learning analytics have to be used in order to study them in a flexible and accurate manner. These techniques can be simple, such as the ones usually found in LMS (descriptive statistics). However, more complex analytical techniques can be used, such as machine learning techniques (a subset of artificial intelligence). The latter are analogous to the computational thought of the human brain and operate with what is known as artificial intelligence. Machine learning techniques of classification and clustering [15] are among the most widely applied techniques for data analysis in educational environments. The use of these techniques for the analysis of both student and teacher behaviors will provide the teacher and those responsible for educational institutions with ideas to introduce improvements into the learning environment [11].

In brief, machine learning techniques are used, as these techniques are currently considered to provide the researcher with more data in the field of cognitive psychology and learning than traditional statistical techniques [16,17]. In particular, machine learning techniques permit personalized learning and provide individualized information on the development of student learning. Prediction techniques facilitate early detection of at-risk students and, therefore, personalized [18] help from the teacher [19,20]. Machine learning techniques also provide information on the effects of predicting the independent variable over each of the dependent variables in percent effects [21].

### 1.3. Design of the Blended-Learning Space in Nursing Instruction

Blended teaching, increasingly present in educational scenarios, is done through a blend of face-to-face (F2F) and virtual learning on LMS, known as blended learning. However, there is no generalized agreement on the taxonomy of blended learning [22]. Nevertheless, its differences with blended learning are accepted; in the blended learning environment, the student completes 80% with LMS and 20% is F2F, hereafter referred to as Blended Learning type 1. In contrast, blended learning (80% interaction in the LMS) is a space where feedback is done 80% of the time through F2F and 20% through LMS [18,23], hereafter referred to as Blended Learning type 2. Recent investigations have found that the replacement blended environment accompanied by the use of active methodologies (e.g., PBL, use of hypermedia resources, flipped learning experiences, and quizzes, or all at once) improved the learning results of students [3,24]. These achievements are especially significant in university environments [25,26] because future graduates will have to develop collaborative work skills, problem-solving independence, and the use of new technologies. These skills are essential for good development of entrepreneurship.

Along these lines, recent studies have shown that [27] an educational intervention that applies blended learning methodology can easily be added into nursing curricula. This type of learning enhances learning in this field. Recent systematic research indicated that blended learning together with PBL is a methodology that ensures effective learning among nursing students [28]. This type of paradigm is more effective than traditional teaching such as face to face. The reasons are that students need to develop the knowledge and skills necessary in clinical practice. Several studies recommend nursing teachers to use multifaceted techniques (blended learning, learning based in projects, etc.) to promote effective learning beyond face-to-face teaching [29]. While these studies highlight the need to train teachers in these techniques [28], the main reason is that, traditionally, teaching has been done face to face, and an organized transfer towards the use of these methodological resources is needed. A recent systematic review showed that, since 2018, there has been a growing interest in the implementation of these experiences in nursing studies. However, an increase in these experiences and more research in this discipline of knowledge are needed [30].

Moreover, blended learning environments permit an evaluation of the whole teaching–learning process in a systematic and simple way. Thus, the suggestion is that there are different variables that influence successful learning in this line of investigation into e-evaluation models, especially the learning strategies employed by the students themselves [31,32], the environment in which the learning takes place [33], the teaching design that the teacher brings to the class [30], and the behavioral learning of the students in the LMS [23]. The prediction interval of these variables is situated around 56%-61% [34].

### 1.4. Extraction and Analysis of Information on the Teaching–Learning Process Recorded in LMS

As we have mentioned earlier, development of the teaching through LMS will facilitate the student in learning recording and follow-up behaviours [35]. Many of these learning managers use supervised machine learning techniques, techniques such as multiple regression analysis (MRA), neural network, and SVM. Those techniques help with the detection and subsequent prediction of successful and risk behaviours. Behaviours of the students in LMS that have been related to successful learning are, among others [23]:

- the time that is used in carrying out the tasks;
- student time expended on studying theoretical content;
- the results in the self-evaluation test (quiz efforts);
- the quality of forum discussions (type and length of message);
- time employed in analysing the feedback given by the teacher;
- the number and type of messages sent;
- the frequency of access to LMS;
- contribution to content creation;
- files opened; and
- delivery time of the activities.

Therefore, the frequency and systematicness of student interactions and their interactions with LMS are directly related to effective learning [36]. Along these lines, recent investigations [23] have revealed differences in predicting learning results in relation to the variable “teaching methodology” (understood in terms of the pedagogic structure of the teaching, the evaluation procedures, and feedback). The type of activities and the evaluation tests (quizzes, tests, projects, presentations…) are understood to determine the effectiveness of behavioural learning logged on the LMS.

As previously mentioned, application of machine learning techniques to study the logs will allow the teachers to analyze the behavioural learning of their students and to detect at-risk students. In these cases, early intervention will presumably improve student learning responses. Recent studies have confirmed [17,35] that following up with student behavioural learning in the LMS facilitates the identification of at-risk students with an explained variance of 67.2%.

In summary, the use of machine learning techniques will permit the study of behavioral learning of both students and teachers on the platform, which will facilitate the application of prediction techniques to the learning results [37]. Reviewing the investigations presented earlier, we consider it important to study the behavior of PBL in the LMS. As has been indicated, there are few studies in that field, and more information is needed that will help to improve teaching practices in these environments [38]. Project work and personalization of learning in LMS have been proven to have significant effects on the quality of learning. Particular relevance has been in Health Science degrees, such as nursing or medicine, etc., since it facilitates work on clinical cases in a collaborative way and optimizes the results applied to real learning contexts [39].

This research study was performed to analyze data of students’ online and face-to-face (F2F) activity in a blended nursing learning course. We applied two types of blended learning: Blended Learning type 1 [in which the interaction between the teacher and students is 80% in the LMS and 20% Face to Face (F2F)] and Blended Learning type 2 [in which the interaction between the teacher and students is 20% in the LMS and 80% Face to Face (F2F)].

In light of the above, the hypotheses in this study were the following:

H 1: The types of blended learning (Blended Learning type 1 vs. Blended Learning type 2) used will predict student learning outcomes;

H 2: The types of blended learning (Blended Learning type 1 vs. Blended Learning type 2) used will predict the learning behaviours logged on the LMS; and

H 3: The type of clusters will be different for each type of blended learning used (Blended Learning type 1 vs. Blended Learning type 2).

## 2. Materials and Methods

### 2.1. Design

A quasi-experimental post-treatment design with an equal control group (in terms of metacognitive skill) was used. Likewise, learning outcomes (learning outcomes in the development of project-based learning; learning outcomes in exhibition of project-based learning; learning outcomes in the test; and learning outcomes total) and behavioral learning in the LMS were the dependent variables (access to complementary information; Access to guidance to prepare PBL; Access to theoretical information; Access to teacher feedback; and mean visits per day).

### 2.2. Participants

A sample of 120 university students was assembled following the third year of their nursery degree in Spain (the degree has four years) during one semester (9 weeks): 63 followed the Blended Learning type 1 methodology and 57 followed the Blended Learning type 2 methodology (see Table 2). The students were assigned to each blended learning group (Blended Learning type 1 vs. Blended Learning type 2) by means of convenience sampling. The work was developed in the subject of “Quality management methodology of nursing services.”

### 2.3. Instruments

a. *LMS UBUVirtual version 3.1*. A Moodle-based learning management system (LMS) was used that began with a constructivist approach and was developed through a modular system. It is a personalized Moodle-based LMS. An LMS is a modular learning environment that permits interaction and feedback between teacher and students, in many cases in real time, and in addition it facilitates the process of automated feedback.

b. The (ACRAr) Scales of Learning Strategies by Román & Poggioli [40]. This widely tested instrument identifies 32 strategies at different points in the information processing cycle. The reliability indicators on the scale were between α = 0.75 to α = 0.90 and the indicators of content validity were between r = 0.85 and r = 0.88. The subscale of metacognitive skills was applied in this study; this scale incorporated 17 strategies about the use of metacognitive skills into the problem solving tasks. A reliability index of α = 0.80 was obtained in this study; the reliability indicator on this subscale was α = 0.90 and the indicator of validity was r = 0.88.

c. *Student learning results: the results were recorded in the different evaluation procedures*. (1) Multiple-choice tests on the theoretical contents of the subject (test) were assigned a weight of 30% of the final grade. The test had 10 multiple-choice questions (four possible answers) with only one correct response. As well, five questionnaire-type quizzes were administered, one for each thematic unit. Cronbach’s Alpha reliability of the test was α = 0.81. (2) Development of PBL, with a weight of 25%, was measured with a rubric, which can be seen in Supplemental Material Table S1. (3) Likewise, the exhibition of the PBL, with a weight of 20%, was also measured with a rubric and can be seen in Supplemental Material Table S2. In the final mark, Cronbach’s Alpha reliability of PBL was α = 0.62. This result is lower because there was less dispersion among the scores in this type of evaluation test. Since the performance of the groups was quite uniform, this aspect can be checked in the results section and it is in accordance with the philosophy of PBL. Finally, the learning outcomes total covered the weighted scores of all the results (over 10 points). 4) The students solved five practices, and this part was 25% of the final grade. However, in this part all students had the highest qualification since the teacher reviewed the practices continuously, and if they were not correct the teacher ordered them to be repeated. Therefore, because it is not discriminate it has not been included in the analysis. Examples of the PBLs developed can be found at this link https://riubu.ubu.es/handle/10259/3753/discover. 

### 2.4. Procedure

Convenience sampling was followed for the choice of the sample. This was due to the possibility of working with this methodology by a specialist teacher who attended to both groups, and in this way the "type of teacher" effect was avoided. Before the instructional intervention, the two groups (Blended Learning type 1 vs. Blended Learning type 2) were scored on the metacognitive skills Scale of ACRAr [40], with the aim of establishing the similarities between both groups in terms of metacognitive skills.

As stated in the introduction, Blended Learning type 1 was applied to the experimental group, a learning environment in which the interactions between teacher and student were 20% F2F and 80% LMS. Likewise, Blended Learning type 2 was applied to the control group, a learning environment in which the interactions between teacher and students were 20% LMS and 80% F2F. In the experimental Group, hypermedia resources were used such as videos, and feedback was through the LMS. In contrast, classroom interactions between teacher and students and feedback in the control group were all F2F. In both groups, PBL methodology was followed. The difference, as has been pointed out, consisted of the type of blended learning in use (Blended Learning type 1 vs. Blended Learning type 2). Project development was done in both (the control and the experimental) groups in a collaborative way. The project work was completed in small groups of students of between 2 and 5 members.

### 2.5. Data Analysis

The following statistical analyses were applied: (1) Analysis of asymmetry and kurtosis; (2) analysis of the variance of a fixed-effect factor (ANOVA); (3) multiple regression analysis (MRA) [appropriate Tolerance (T) values were considered close to one and, with respect to the variance inflation factor, the values were between 1–10]; (4) cluster analysis. Package for the Social Sciences (SPSS) v.24 was used to perform the different analyses [41]. Likewise, the Goodness-of-fit indices were measured by structural equation modeling (SEM) and was used to study the settings of the machine learning technique to predict the learning results. The calculations were performed with the Statistical Package for the Social Sciences (SPSS) AMOS v.24 [42]. (5) Finally, to visualize the results in a cluster analysis, RapidMiner Studio software [43] was used.

### 2.6. Ethical Considerations

The research project was approved by the Ethics Committee of the University of Burgos. Previously, at the start of the project, the students were informed of the objectives, and their participation was at all times on a voluntary basis. Likewise, informed consent of each participant was recorded in writing.

## 3. Results

### 3.1. Previous Statistical Normalcy Analysis in the Sample

Before starting the research, the indicators of normality were studied. The results obtained from earlier statistical analyses with regard to the normality of the sample are presented below (values higher than |2.00| indicate extreme asymmetry, the lowest values indicate normality, and the values of between |8.00| and |20.00| suggest extreme kurtosis [44]). The results of metacognitive skills on the ACRAr subscale in both groups were acceptable for both indicators (see Table 3). Therefore, parametric statistics were used. Descriptive statistics are also shown in Table A1 and Table A2 (see Appendix A).

### 3.2. Previous Statistical Analysis of Homogeneity between the Groups before the Intervention

Significant differences between both groups (experimental and control) in their use of metacognitive strategies were anlayzed before application of the different types of blended learning (Type 1 vs. Type 2). To do so, a single-factor ANOVA with fixed-effects was performed (blended learning type) on the results. No significant differences were found between both, so they can be considered similar groups (F_1_, _119_ = 0.276; *p* = 0.601; η^2^ = 0.002) in the ACRAr subscale of metacognitive skills.

Similarly, in order to study which type of supervised learning technique would be the most appropriate, the Goodness-of-fit indices were measured in the structural equation modeling (SEM) that was used to study the settings of the machine learning technique to predict the learning results. The calculations were performed with the Statistical Package for the Social Sciences (SPSS) AMOS v.24, as may be seen in Table 4, and no dependent relations between the observed values and the different prediction methods (LR, DT, RBFN, and kNN) were found for any of the four prediction models. Among these possibilities, the following were applied in the MRA.

### 3.3. Hypothesis 1.

MRA was performed to study the predictive value of the variable blended learning type applied to the student learning outcomes. An *R*^2^ = 0.404 was found, which indicates that this variable explained 40.04% of the variance in the learning results. The Tolerance (*T)* values were within an interval of 0.106 and 0.336 and the Variance Inflation Factor (*VIF*) between 3.491 and 9.45, so none of the variables had to be removed. Likewise, the highest partial correlation was found in the Learning Outcomes Total (*r* = 0.586; *p* = 0.000), see Table A3.

### 3.4. Hypothesis 2.

MRA yielded a figure of *R^2^* = 0.719 in the study of the predictive value of blended learning applied to student behaviors on the platform. This figure indicated that the blended learning type in use explained 71.19% of the variance in the learning behaviors of students on the platform. The Tolerance (T) values were situated within an interval between 0.136 and 0.539 and the Variance Inflation Factor (VIF) between 1.472 and 7.346, so that no variable had to be removed. The highest partial correlation was found in Access to Teacher Feedback (*r* = 0.448), see Table A4.

### 3.5. Hypothesis 3.

A k-means clustering technique was applied in each type of blended learning in use (Blended Learning type 1 vs. Blended Learning type 2), as seen in Table 5. Three clusters are shown in Table 5 that were found in the two types of blended learning (Cluster 1, Sufficient; Cluster 2, Intermediary; and Cluster 3, Excellent. The classification of Cluster type was according to the maximum possible value in each learning outcome and number of accesses obtained). Higher values for performance were found in the Blended Learning type 1 rather than the Blended Learning type 2 in all three clusters, specifically in Learning Outcomes Total. Likewise, with regard to the learning behaviors developed by students in the type of blended learning in use (Blended Learning type 1 vs. Blended Learning type 2), as may be seen in Table 5, a higher number of log-ons to the platform in the Blended Learning type 1 rather than the Blended Learning type 2 environment were found, except for student queries on theoretical information provided by the teacher (see Table 6).

**Figure 1:**
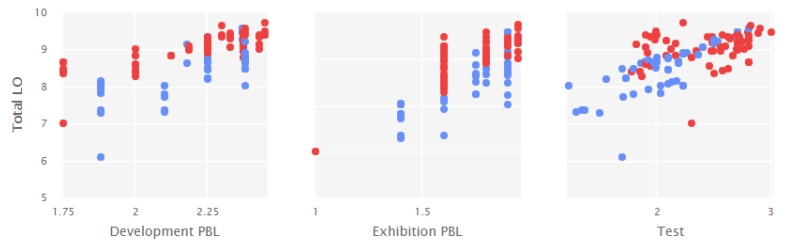
Distribution of scores in the different types of Learning outcomes. Note. Development PBL = Development Project-Based Learning outcomes; Exhibition PBL = Exhibition Project-Based Learning outcomes; Test = Test Learning outcomes; Total LO = Learning outcomes Total.

**Figure 2:**
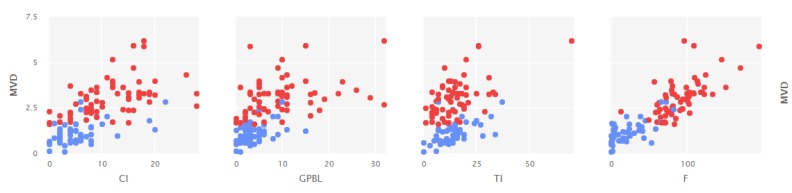
Distribution of scores in the different types of behavioral learning in the LMS. Note. CI = Access to Complementary Information scores; CPBL = Access to guidance to prepare PBL scores; TI = Access to Theoretical Information scores; F = Access to Teacher Feedback; MVD = Mean Visits per day.

Figure 1 shows the scores in the two groups: experimental group (red color) and control group (blue color). As can be seen, there was a greater homogeneity of higher scores in the experimental group for different types of Learning outcomes. Similarly, Figure 2 points to the distributions of LMS behavioral learning scores in different resources.

## 4. Discussion

In the blended learning environments, the type of teaching design appears to be a predictive factor in both the learning results and the learning behaviors that the students develop in the LMS. blended learning with 80% of interactions in LMS appeared to be more effective, both with respect to the learning results of the students and the effectiveness of the learning behaviors that they develop. This type of pedagogic design includes the use of hypermedia resources that strengthen teacher feedback in real time, which furthers the development of SRL strategies [24,35]. This aspect is of special relevance for teachers in nursing higher education, and the implicit message is that they would be well advised to design their materials for use in a blended learning environment [27,28,29,30], as those environments appear to have increased the effectiveness of active methodologies, especially PBL with hypermedia resources in LMS [38]. In addition, Blended Learning type 1 (80% the interaction in the LMS) strengthens students’ use of learning-based projects that have been considered more effective in the LMS [2,23]. These behaviors range from access to feedback given by the teacher to tasks carried out by the student or the collaborative groups and the average number of visits per day [23]. All of this indicates that the Blended Learning type 1 design increases the interaction of the student in the LMS and that interaction also facilitates student access to feedback from the teacher, as the LMS can be consulted as many times as necessary when learning, an aspect that is less feasible with F2F instruction [9,10,18]. In this way, the teachers can structure their help and prepare specific materials for each group.

In addition, machine learning techniques have been used in this study, in view of their effective use with what is known as data mining [13,18,36]. In particular, supervised and unsupervised machine learning techniques have been used (linear regression and clustering k-means methods, respectively). Prediction and clustering studies, among others, can be conducted with these techniques, which help the teacher to gain knowledge of the learning characteristics of students and to predict at-risk students [23,34,38]. Even so, it is true that these techniques should be used throughout the whole teaching process to be able to develop personalized actions for student learning [35,36]. In subsequent studies, therefore, development of the learning process among students at the start, in the middle, and at the end of the study module will be analyzed with machine learning techniques [12,13].

## 5. Limitations

This study has limitations, but the results of this study should, nevertheless, be given prudent consideration. Limitations include the following: methodological intervention was in one university, the students were from a specific country, convenience sampling was applied, the knowledge area of the students was specific, and the type of design (quasi-experimental) was also specific. Although, it must be taken into consideration that there are few specific studies to test the effectiveness of this type of methodology in nursing students. Studies that have been carried out have similar characteristics that are justified from the specificity of this research [28,29,30].

Therefore, future studies will be directed at increasing the size of the sample and the diversity of the nursing degree course level. Therefore, this profession is subject to continuous theoretical and technological advances that require systematic research on how to teach better in order to learn more effectively.

## 6. Conclusions

This research study has identified the characteristics to design an effective LMS in the nursing degree. The use of prediction and clustering techniques is very important to facilitate personalized learning and to analyze how resources are better utilized in the blended learning space. This type of analysis can be automatically generated in LMS environments, such as Moodle, and could be integrated in modules and plugins. These tools would facilitate rapid and straightforward generation of those analyses, which would be of great utility for the teacher and would assist with the early detection of at-risk students, as well as behavioral analyses of both the individual student and the collaborative groups of students, which would foreseeably increase the teaching quality and learning outcomes. This need has been underlined in such studies as those by Peña-Ayala [12] and Romero et al. [17], and they have to be approved by university management, but they will virtually be a necessity in 21st century teaching as we move closer to personalized on-line teaching, both in F2F teaching and in virtual learning environments. In summary, this teaching design is especially significant in the nursing degree since project work is a practice that has proved very effective in the training of future professionals.

Good results have been obtained in all assessment tests in the two types of blended learning. However, the type of blended learning that applied automated feedback and hypermedia resources obtained even better results (more percentage of work in the LMS) [28,29,30]. One explanation may be that the student can access information in the LMS at any time, which is not possible for F2F interaction, and this facilitates personalization of learning and motivates the student [7,8,39]. Therefore, incorporating these forms of work in teaching in the field of health is a very effective option.

The results obtained are in line with those found in the research of Oh & Lee [28]. The use of PBL methodology in blended learning environments empowers nursing students to acquire practical skills that are of great help for nursing work in real intervention environments [28]. This form of teaching is flexible [30] because it facilitates development and tests hypotheses in the resolution of tasks similar to those they will encounter in a working environment, and in addition, group work facilitates the acquisition of collaborative work skills, which they will also encounter in such working environments. All this increases the self-efficacy and critical thinking skills of these professionals. Recent studies recommend the application of this methodology within the nursing degree curricula [27].

In sum, it can be concluded that this way of teaching seems to be effective for nursing students. Although, more studies are needed in this field aimed at studying the effectiveness of blended learning in teaching in the nursing degree. 

## Figures and Tables

**Table 1:** Preliminary elements to take into account for the design of learning activities.

Questions for Activities Design	Subject Module Design (Teacher)	Aspects to Evaluate (Teacher and Students)
What	What is the object of the learning process? What competences are to be developed in the students?	Learning goals. Design of Knowledge.
How	Design of learning tasks.	Test its effectiveness for the achievement of the proposed learning aims
Who	To whom is it directed? Gain knowledge of the characteristics of the students.	In the students ✓ Prior knowledge of the learning material. ✓ Metacognitive skills that the teacher employs.
When and Where	Chronogram of timing of the tasks and the moments and spaces in which they will take place.	Teacher Gradual sequencing of the difficulty of the learning tasks.
		✓ Planning of process-oriented feedback in each of the learning experiences.
	Behavior of (individual and group) learners on the platform.	✓ Evaluation of student behavior in the various activities that have been designed and in (individual and group) teacher feedback through the platform.

**Table 2:** Group assignment and descriptive statistics for age, *^a^n* = 60. *^b^n* = 62.

Groups	Men			Women		
	*n*	*M_age_*	*SD_age_*	*n*	*M_age_*	*SD_age_*
Experimental Group, Blended Learning type 1 (^a^n)	7	23.29	2.56	56	22.30	2.13
Control Group, Blended Learning type 2 (^b^n)	9	24.67	4.12	48	23.83	5.13

Note. *M_age =_* Mean Age; *SD_age_* = Standard Deviation.

**Table 3:** Indicators of asymmetry and kurtosis in the experimental group and control group.

Blended Learning Type 1 (Experimental Group)						Blended Learning Type 2 (Control Group)					
*M*	*SD*	*A*	*ASE*	*K*	*SEK*	*M*	*SD*	*A*	*AES*	*K*	*SEK*
80	23.85	−0.464	0.441	−0.973	0.858	75	28.75	−0.333	0.306	−0.957	0.604

Note. *M* = Mean Age; *SD* = Standard Deviation; *A* = Asymmetry*; K* = Kurtosis; *ASE* = Asymmetry Standard Error; *SEK* = Kurtosis Standard Error.

**Table 4:** Goodness-of-fit indices.

Goodness of Fit Index	LR	RBFN	kNN	Accepted Value
df	5	5	5	
χ^2^	174.121 (*p* = 0.000)	98.279 (*p* = 00.00)	106.532(*p* = 0.00)	*p* > 0.05 α = 0.05
RAMSEA	0.769	0.616	0.683	>0.05–0.08
RAMSEA interval	0.722–0.817	0.568–0.664	0.636–0.732	
SRMR	0.1602	0.1086	0.1152	>0.05–0.08
TLI	0.000	0.000	0.000	0.85–0.90<
CFI	0.000	0.000	0.000	0.95–0.97<
AIC	730.199	474.186	580.261	The lowest value
ECVI	6.085	3.956	4.836	The lowest value
ECVI interval (90%)	5.382–6.849	3.960–4.574	4.214–5.518	The lowest value

Note. df = degrees of liberty; χ^2^ = Chi squared; LR = Linear Regression; DT = Decision Trees; RBFN = Radial basis function network; kNN = k-Nearest Neighbor classification; NFI = normed-fit-index; RMSEA = Root-Mean-Square Error of Approximation; SRMR = Standardized Root-Mean-Square Residual; TLI = Tucker–Lewis index; CFI = comparative fit index; AIC = Akaike Information criterion; ECVI = parsimony index.

**Table 5:** Centers of final clusters for the learning results variable in Blended Learning type 1 and type 2, Blended Learning type 1: ^a^*n* = 1; ^b^*n* = 45; ^c^*n* = 17; Blended Learning type 2: A lost value is observed. *^a^n* = 9; *^b^n* = 30; *^c^n* = 18

	Maximum	Cluster 1 Sufficient	Cluster 2 Intermediary	Cluster 3 Excellent
**Blended Learning type 1**				
Learning outcomes in PBLD	2.50	1.75	2.00	2.34
Learning outcomes in PBLE	2.00	1.00	1.62	1.80
Learning outcomes in test	3.00	2.30	2.24	2.50
Learning outcomes Total	10	7.00	8.62	9.26
**Blended Learning type 2**				
Learning outcomes in PBLD	2.50	1.88	2.09	2.32
Learning outcomes in PBLE	2.00	1.50	1.59	1.87
Learning outcomes in test	3.00	1.70	1.82	2.39
Learning outcomes Total	10	6.08	8.00	9.08

Note. PBLD = Project-Based Learning Development; PBLE = Project-Based Learning Exhibition.

**Table 6:** Centers of final clusters and the variable behavioral learning on the LMS in Blended Learning type 1 and type 2. Blended Learning type 1: a lost value is observed. *^a^n* = 31; *^b^n* = 27; *^c^n* = 5; Blended Learning type 2: Two lost values were observed. *^a^n* = 36; *^b^n* = 16; *^c^n* = 6.

	Interval	Cluster 1 Sufficient	Cluster 2 Intermediate	Cluster 3 Excellent
**Blended Learning type 1**				
Access to Complementary Information	0–14	9	14	14
Access to guidance to prepare PBL	0–6	10	9	6
Access to Theoretical Information	0–14	12	18	14
Access to Teacher Feedback	0–158	69	103	158
Mean Visits per day	0–7	2.48	3.40	4.51
**Blended Learning type 2**				
Access to Complementary Information	0–7	4	6	7
Access to guidance to prepare PBL	0–5	3	5	5
Access to Theoretical Information	0–14	12	18	14
Access to Teacher Feedback	0–66	5	30	66
Mean Visits per day	0–2	0.84	1.30	1.93

Note; PBL = Project-Based Learning Development.

## Supplementary Materials

The following are available online at https://www.mdpi.com/1660-4601/17/5/1589/s1, Table S1: Rubric to evaluate development of Project-Based Learning, Table S2: Rubric to evaluate Exhibition of Project-Based Learning.

## Appendix A

**Table A1:** Descriptive statistics for the Learning outcomes.

Learning Outcomes	Blended Learning Type 1 (Experimental Group)					Blended Learning Type 2 (Control Group)				
	*Minimum*	*Maximum*	*M*	*SD*	α	*Minimum*	*Maximum*	*M*	*SD*	α
Learning outcomes in the preparation of PBL	1.75	2.45	2.23	0.21	0.79	1.88	2.38	2.25	0.17	0.81
Learning outcomes in presentation of PBL	1.00	1.95	1.74	0.17	0.76	0	1.90	1.75	0.19	0.75
Learning outcomes in test	1.78	3.00	2.43	0.35	0.80	1.23	2.80	2.17	0.42	0.78
Learning Outcomes Total	7.00	0.72	9.03	0.44	0.73	6.08	9.57	8.64	0.66	0.70

Note: *M* = Mean; *SD*= Standard Deviation; PBL = Project-Based Learning; *α* = Reliability index of Cronbach’s Alpha.

**Table A2:** Descriptive statistics for Behavioral learning in the LMS.

Behavioral Learning in the LMS	Blended Learning Type 1 (Experimental Group)				Blended Learning Type 2 (Control Group)			
	*Minimum*	*Maximum*	*M*	*SD*	*Minimum*	*Maximum*	*M*	*SD*
Access to Complementary Information	0	28	5.07	11.60	0	22	5.07	4.60
Access to Guidance for the Preparation of PBL	0	32	9.46	7.59	0	15	3.60	3.01
Access to Theoretical Information	1	70	14.84	10.23	0	37	13.81	7.49
Access to Teacher Feedback	0	194	90.90	29.21	0	82	18.43	21.14
Mean Visits per day	0.41	6.15	3.04	1.02	0.06	2.81	1.08	0.58

Note: *M* = Mean; *SD* = Standard Deviation

**Table A3:** Coefficients in the prediction of learning outcomes with variable types of Blended Learning.

	Unstandardized Coefficients		Standardized Coefficients	*t*	*p*	Correlations			Collinearity Statistics	
	*B*	*Standard Error*	*Beta*			*Zero Order*	*Partial*	*Part*	*Tolerance*	*VIF*
(Constant)	−2.52	0.64		−3.93	0.00					
Learning outcomes Elaboration PBL	−1.22	0.32	−0.48	−3.87	0.00	0.01	−0.34	−0.28	0.34	3.00
Learning outcomes PBL Exhibition	−2.29	0.37	−0.82	−6.18	0.00	−0.06	−0.50	−0.44	0.29	3.49
Learning outcomes in test	−0.69	0.17	−0.56	−4.00	0.00	0.32	−0.35	−0.28	0.26	3.91
Learning outcomes Total	1.39	0.19	1.64	7.49	0.00	0.33	0.57	0.53	0.11	9.45

Note: Dependent variable: Blended Learning type; PBL = Project-Based Learning; *VIF* = Variance Inflation Factor.

**Table A4:** Coefficients in the prediction of Behavioral learning with variable types of Blended Learning.

	Unstandardized Coefficients		Standardized Coefficients	*t*	*p*	Correlations			Collinearity Statistics	
	*B*	*Standard Error*	*Beta*			*Zero Order*	*Partial*	*Part*	*Tolerance*	*VIF*
(Constant)	1.02	0.05		19.53	0.00					
Access to Complementary Information	0.002	0.01	0.03	0.38	0.71	0.51	0.035	0.02	0.54	1.85
Access to guidance for the Preparation of PBL	0.02	0.01	0.20	3.14	0.002	0.45	0.28	0.15	0.60	1.66
Access to Theoretical Information	−0.01	0.003	−0.21	−3.49	0.001	0.06	−0.31	−0.17	0.68	1.47
Access to Teacher Feedback	0.01	0.001	0.60	5.37	0.00	0.82	0.45	0.26	0.19	5.25
Mean Visits per day	0.08	0.05	0.20	1.55	0.13	0.76	0.14	0.08	0.14	7.35

Note: Dependent variable: Blended Learning type; *VIF* = Variance Inflation Factor.
