# Supplementary material for: Effectiveness of Blended Learning in Nursing Education
Source: Int J Environ Res Public Health. 2020 Mar 1;17(5):1589. doi: 10.3390/ijerph17051589 (PMC7084479; doi:10.3390/ijerph17051589)
Supplement: Supplementary file 1 [file ijerph-17-01589-s001.zip › ijerph-708854-supplementary.docx]

**Supplemental Material**

**Table S1.** Rubric to for the evaluation developmental of PBL.

The global score for project developmental as presented in the teaching guide of the study module was 25%. The points are distributed below for each criterion in the evaluation with respect to the overall score. The evaluation criteria were designed following the updated version of the taxonomy of Bloom ([Anderson,](http://discoverlibrary.vanderbilt.edu/primo_library/libweb/action/search.do?vl(freeText0)=Anderson%2c+Lorin+W.&vl(78204569UI0)=creator&vl(63907227UI1)=all_items&vl(1UIStartWith0)=exact&fn=search&tab=acorn&mode=Basic&vid=VANDERBILT&scp.scps=scope%3a(vanunicorn)&ct=lateralLinking) [Krathwohl](http://discoverlibrary.vanderbilt.edu/primo_library/libweb/action/search.do?vl(freeText0)=Krathwohl%2c+David+R.&vl(78204569UI0)=creator&vl(63907227UI1)=all_items&vl(1UIStartWith0)=exact&fn=search&tab=acorn&mode=Basic&vid=VANDERBILT&scp.scps=scope%3a(vanunicorn)&ct=lateralLinking) & [Bloom, 2001).](http://discoverlibrary.vanderbilt.edu/primo_library/libweb/action/search.do?vl(freeText0)=Bloom%2c+Benjamin+S.+(Benjamin+Samuel)%2c+1913-1999+Taxonomy+of+educational+objectives.&vl(78204569UI0)=creator&vl(63907227UI1)=all_items&vl(1UIStartWith0)=exact&fn=search&tab=acorn&mode=Basic&vid=VANDERBILT&scp.scps=scope%3a(vanunicorn)&ct=lateralLinking)

| **Evaluation criteria** | **Excellent** | **Very good** | **Good** | **Hardly acceptable** | **Insufficient** |
| --- | --- | --- | --- | --- | --- |
| **1. Uses up-to-date bibliographic references from scientific sources.** | Uses up-to-date bibliographic references, from the current course or from the past four years, (80 to 100%) in relation to the chosen topic of the project. | Uses up-to-date bibliographic references, from the current course or from the past four years, (60 to 79%) in relation to the chosen topic of the project. | Uses up-to-date bibliographic references, from the current course or from the past four years, (40 to 59%) in relation to the chosen topic of the project. | Makes no use of up-to-date bibliographic references, from the current course or from the past four years (less than 30 %). | Makes no use of up-to-date bibliographic references, from the current course or from the past four years (less than 30 %). |
| **5%** | **5** | **4** | **3** | **1-2** | **0** |
| **2. Applies APA or Vancouver norms in a precise and careful way and the written document reflects a precise and careful use of spelling and punctuation. Using a writing style in the third person singular.** | Applies APA or Vancouver norms in a precise and careful way and the written document reflects a precise and careful use of spelling and punctuation, using a writing style in the third person singular. | Applies APA or Vancouver norms in a precise and careful way and the written document implies careful use of spelling and punctuation, using a writing style in the third person singular. | Applies APA or Vancouver norms with no significant errors and the written document reflects conventional spelling and punctuation, using a writing style in the third person singular with no significant errors. | Mistakes are appreciated in the application of APA or Vancouver norms. Mistakes are detected in the written document (in spelling norms, punctuation signs, and in the use of the subject. | Significant errors are appreciated in the use of APA or Vancouver norms. Significant errors are detected in the written document. |
| **5%** | **5** | **4** | **3** | **1-2** | **0** |
| **3. Shows a logical structuring of the chapters that are explained in a precise and systematic organization of the contents and of the proposed intervention, using the conceptual, procedural, and attitudinal contents seen in the study module.** | The structure of the chapters follows a logical line that is explained in a precise and systematic organization of the contents and of the proposed intervention, using the conceptual, procedural and attitudinal contents seen in the study module. | The structure of the chapters follows a logical line that is explained in a systematic organization of the contents and of the proposed intervention, using the conceptual, procedural and attitudinal contents seen in the study module | The final conclusions of the project and the future lines of work imply a good degree of preparation, evaluation and reflection. | The final conclusions of the project and the future lines of work present weaknesses in its preparation, in the evaluation and in its reflection. | The final conclusions of the project and the future lines of continuity imply a good degree of preparation, evaluation and reflection. |
| **5%** | **5** | **4** | **3** | **1-2** | **0** |
| **4. Argues the final conclusions of the project and the future lines of work imply a degree of preparation, evaluation, and reflection that is both precise and careful.** | The final conclusions of the project and the future lines of work imply a degree of preparation, evaluation, and reflection. that is both precise and careful | The final conclusions of the project and the future lines of work imply a degree of preparation, evaluation, and reflection that is careful. | The final conclusions of the project and the future lines of work imply a good degree of preparation, evaluation, and reflection. | The final conclusions of the project and the future lines of work present weaknesses in their preparation, evaluation, and reflection. | Significant errors are appreciated in the conclusions and in the future lines of work. |
| **10%** | **5** | **4** | **3** | **1-2** | **0** |

**Table S2.** Rubric to for the evaluation Exhibition of PBL.

The global score for project preparation as presented in the teaching guide of the study module was 20%. The points were distributed below for each criterion in the evaluation with respect to the overall score. The evaluation criteria were designed following the updated version of the taxonomy of Bloom ([Anderson,](http://discoverlibrary.vanderbilt.edu/primo_library/libweb/action/search.do?vl(freeText0)=Anderson%2c+Lorin+W.&vl(78204569UI0)=creator&vl(63907227UI1)=all_items&vl(1UIStartWith0)=exact&fn=search&tab=acorn&mode=Basic&vid=VANDERBILT&scp.scps=scope%3a(vanunicorn)&ct=lateralLinking) [Krathwohl](http://discoverlibrary.vanderbilt.edu/primo_library/libweb/action/search.do?vl(freeText0)=Krathwohl%2c+David+R.&vl(78204569UI0)=creator&vl(63907227UI1)=all_items&vl(1UIStartWith0)=exact&fn=search&tab=acorn&mode=Basic&vid=VANDERBILT&scp.scps=scope%3a(vanunicorn)&ct=lateralLinking) & [Bloom, 2001).](http://discoverlibrary.vanderbilt.edu/primo_library/libweb/action/search.do?vl(freeText0)=Bloom%2c+Benjamin+S.+(Benjamin+Samuel)%2c+1913-1999+Taxonomy+of+educational+objectives.&vl(78204569UI0)=creator&vl(63907227UI1)=all_items&vl(1UIStartWith0)=exact&fn=search&tab=acorn&mode=Basic&vid=VANDERBILT&scp.scps=scope%3a(vanunicorn)&ct=lateralLinking)

| **Evaluation criteria** | **Excellent** | **Very good** | **Good** | **Hardly acceptable** | **Insufficient** |
| --- | --- | --- | --- | --- | --- |
| **1. Develops a proposal and an organizational plan for the presentation of the project and adjusts it to the time that is allowed for the exhibition (10´).** | The organization of the project defense is well-structured, and it follows a precise line of argument that provides a concise summary of the work. It is within the time limit (10´) for the defense of the project. | The organization of the project defense is well-structured, and it follows a precise line of argument that provides a concise summary of the work. It is within the time limit (10´) for the defense of the project. | The organization of the project defense is structured. It is within the time limit (10´) for the defense of the project. | The organization of the project defense has problems structuring the presentation of the content of the work. It is not well-adjusted to the time limit (10´) for the defense of the project. | Significant problems are detected with the structure of the defense. It is not well-adjusted to the time limit (10´) for the defense of the project. |
| **5%** | **5** | **4** | **3** | **1-2** | **0** |
| **2. Develops a defense with precise and appropriate intonation. A technical vocabulary is used and it is in keeping with the object of the exhibition.** | Appropriate and precise intonation is employed. A technical vocabulary is used, and it is in keeping with the object of the presentation. | Appropriate and precise intonation is employed that is in keeping with the object of the presentation. | The intonation presents some non-significant errors in precision that hinder an understanding of the discourse. | The intonation of the speech presents errors of precision and appropriateness. A high percentage of non-technical vocabulary is employed (60%-to- 100%). | The intonation is unsteady and is an obstacle to the development of the discourse.  It employs colloquial vocabulary. |
| **5%** | **5** | **4** | **3** | **1-2** | **0** |
| **3. Argues responses to the questions raised by the teacher and/or the companions on the project.** | The reasoning is certain and precise. The responses are prepared and involve a deep mastery over the subject of the project. Uses procedure and expresses attitudes seen on the course. | The reasoning is certain and precise. The responses are prepared and involve a deep mastery over the subject of the project. Uses procedure and expresses attitudes seen on the course. | The reasoning is correct. The responses are prepared and imply acceptable knowledge of the project topic. | The reasoning is doubtful in response to the questions that are advanced. Although the responses are in keeping with the object of the project. | The reasoning is doubtful to the questions that are advanced. The responses are ambiguous and general. |
| **5%** | **5** | **4** | **3** | **1-2** | **0** |
| **4. Argues the final conclusions of the project and the lines of future continuity imply a degree of preparation, evaluation and reflection that is precise and careful.** | The resources in support of the presentation were used skillfully and care was taken over their preparation. It has all facilitated the didactic aspects of the presentation. | The resources in support of the presentation were skillfully used. Care was taken over their preparation. It has all facilitated the didactic aspects of the presentation. | The resources in support of the presentation were correctly used. | The resources in support of the presentation were not used skillfully, errors were appreciated in its preparation that complicates the didactics of the presentation. | The resources employed for the presentation were carefully used in its preparation. All of which has significantly complicated the didactics of the presentation. |
| **5%** | **5** | **4** | **3** | **1-2** | **0** |

| 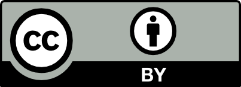 | © 2020 by the authors. Submitted for possible open access publication under the terms and conditions of the Creative Commons Attribution (CC BY) license (http://creativecommons.org/licenses/by/4.0/). |
| --- | --- |
